# A Testbed to Evaluate the FIWARE-Based IoT Platform in the Domain of Precision Agriculture

**DOI:** 10.3390/s16111979

**Published:** 2016-11-23

**Authors:** Ramón Martínez, Juan Ángel Pastor, Bárbara Álvarez, Andrés Iborra

**Affiliations:** Electronics Engineering and Systems Division (DSIE), Universidad Politécnica de Cartagena, Campus Muralla del Mar s/n, 30202 Cartagena, Murcia, Spain; rmartinezcarreras@gmail.com (R.M.); juanangel.pastor@upct.es (J.Á.P.); balvarez@upct.es (B.Á.)

**Keywords:** precision agriculture, wireless sensor networks, Internet of Things, FIWARE

## Abstract

Wireless sensor networks (WSNs) represent one of the most promising technologies for precision farming. Over the next few years, a significant increase in the use of such systems on commercial farms is expected. WSNs present a number of problems, regarding scalability, interoperability, communications, connectivity with databases and data processing. Different Internet of Things middleware is appearing to overcome these challenges. This paper checks whether one of these middleware, FIWARE, is suitable for the development of agricultural applications. To the authors’ knowledge, there are no works that show how to use FIWARE in precision agriculture and study its appropriateness, its scalability and its efficiency for this kind of applications. To do this, a testbed has been designed and implemented to simulate different deployments and load conditions. The testbed is a typical FIWARE application, complete, yet simple and comprehensible enough to show the main features and components of FIWARE, as well as the complexity of using this technology. Although the testbed has been deployed in a laboratory environment, its design is based on the analysis of an Internet of Things use case scenario in the domain of precision agriculture.

## 1. Introduction

The purpose of precision agriculture (PA) is to increase farm productivity by capturing and interpreting data concerning the climate, weather, terrain, water quality and crop status. In recent years, farmers have begun using information systems to improve crop management and increase productivity. These systems include sensors for monitoring the crops and for tracing the products from crops to shelves. As a result, agriculture is becoming a data-intensive industry. In this sense, precision agriculture poses many of the same problems as the other areas where IoT is being developed (industry 4.0, logistics, smart grids, smart cities, etc.), as well as others of its own domain, such as those described in [1,2]: (1) organization and operation of the farm, (2) traceability of products, (3) environmental requirements, (4) decision support to increase farm performance, (5) synchronization between sensors and agricultural machinery, (6) the automation of agricultural tasks, etc.

The biggest challenge for PA is capturing enough high-quality data to make the right decisions. In open spaces, most of the data comes from aerial photographs taken by satellites and aircraft and from sensors and cameras mounted on agricultural machinery and, in recent times, even drones are being used. Precision agriculture calls for a wide array of technologies to capture data, contextualise them in time and space, statistically characterise them, save and merge them and finally analyse them for decision-making purposes. In this scenario, data processing is often based on statistical techniques and artificial vision where no particular attention is paid to latency.

The authors’ research group has quite a lot of experience in wireless sensor networks [3], one of the essential components of the Internet of Things (IoT) [4]. In the agricultural sector we have worked on improving crop and irrigation systems owing to their importance in the semiarid region of Murcia (Spain). Reference [5] presents a novel design for a wireless sensor node designed for precision horticulture applications allowing for the use of precision agriculture instruments. This sensor was integrated into the network of experimental sensors of an ecological horticulture undertaking in the Region of Murcia [6], validated in real operating conditions [7] and compared with other studies [8].

Currently, in situ wireless sensor networks (WSN) are not widespread due to the high price of the sensors and the problem of powering them in places where there are no electrical lines. In the absence of external power sources from which sensor can harvest energy, sensor activity (capturing and transmitting data) must be kept to a minimum However, these limitations may change significantly in coming years thanks to advances in the design of power systems and batteries and price reductions resulting from the mass production of miniaturised sensors. It is therefore possible to imagine a scenario in which many sensors measure parameters related to soil (moisture and nutrient levels), water (salinity) and plants (water stress) are deployed. Capturing, transmitting, storing and processing this volume of information collected by the WSN presents a number of challenges, especially regarding integration technologies, communications, databases and computing. Fortunately there are a number of middleware platforms capable of doing most of the work although only some of these are geared for the IoT. One such middleware technology is called FIWARE [9] whose architecture is still being implemented and whose development is being closely monitored by the authors’ research group [10].

FIWARE is the technology promoted by the European Commission to make possible the IoT in the context of the Future Internet [11] in collaboration with the information and communication technology industry. FIWARE is a powerful and complex technology, but to the authors’ knowledge, there are not works that show how to use this technology in precision agriculture and study its appropriateness, its scalability and its efficiency for this kind of applications.

The aims of this paper are to provide such comprehensible use case, and to check whether FIWARE can be scaled to the same extent as agricultural applications. The answers are important because the selection of a particular platform implies a long-term commitment. We have created a testbed to achieve this goal.

The rest of this paper is structured as follows: Section 2 presents the state of the art. Section 3 provides an overview of the FIWARE platform. The testbed design featured in Section 4 is derived from an IoT use case scenario that applies the FIWARE concepts and components discussed in Section 3. Section 4 also describes the test plan designed to assess the FIWARE platform. Section 5 shows the results obtained. Lastly, Section 6 contains the conclusions drawn from the results and future work related to this research.

## 2. State of the Art

The Internet of Things is the vision of a set of technologies, systems and design principles for connecting things, based on the physical environment, via the Internet. As machine to machine communication is increasing, a new category of applications is emerging that demands evolutionary changes in network infrastructure. The original Internet architecture was not conceived to support communications between billions of devices such as sensors with very limited computational resources. That is why we must embark upon the path towards the *Future Internet* [11] through research and the development of intelligent algorithms, new network paradigms and new services. Along this path we will need to overcome major hurdles [12,13] such as those posed by the things themselves (devices), communications between things, between things and the global Internet and the management, identification and modelling of a massive number of changing and virtually unlimited things.

### 2.1. Devices

In the future there will be an even greater deal of heterogeneity in devices connected to the IoT, both intelligent as well as inert (e.g., simply labelled as Radio Frequency Identification, RFID). Moreover, the vast majority of smart devices lack the computing resources needed to implement the IP protocol stack and this situation will continue in the future for many of the devices used in IoT applications [14]. This is the case of WSNs which consist of devices designed to be deployed in large numbers and multiple environments, including remote regions. For these reasons, maximising lifetime, power consumption and cost of deployment are some of the challenges that these networks present. Specifically, energy is the scarcest resource of WSN nodes and is a decisive factor in the useful life of the network. Thus, energy supply for devices integrated in WSN will increasingly depend on different energy harvesting technologies (solar, thermal, wind, etc.) and on new battery miniaturization and ultra capacitor technologies [15,16,17]. As these technologies mature, WSN will be applicable in a wide range of scenarios that require long-life batteries. Regarding cost, according to an IDTechEx forecast [18], the average price per node will drop from $9 per piece in 2011 to $5 per piece in 2021. Communications merit a separate section.

### 2.2. Communications

The challenge of heterogeneity is also present in communications. The basic infrastructure of the IoT is supported by wired and wireless technologies. The biggest challenges are found in wireless technologies which are classified as:

- Short range (including Personal Area Networks (PAN), Local Area Networks (LAN) and wireless Metropolitan Area Networks(MAN)) mesh networks, RFID, Wi-Fi, etc;
- Long range (via cellular networks, wireless Wide Area Networks (WAN) and pseudo-long-range): Global System for Mobile communications (GSM), combination of Worldwide Interoperability for Microwave Access and Code division multiple access (WiMax CDMA), Wideband Code Division Multiple Access (WCDMA) and other networks, and satellite communication.

Another challenge is the unknown and dynamic topology of WSN due to the variable number of devices connected at any given time. WSN typically use short-range wireless mesh networks as their fundamental communication technique (Zigbee [19], 6LowPAN [20], etc.). This being a low power, low range radio system, in current WSN nodes the IEEE 802.15.4 [21] standard is most commonly used. Connections on the edge of the network are low-fidelity, low speed and data is often lost. In the case of global Internet communications, some devices serve as gateways, implement the IP protocol stack and use long-range cellular networks. Most devices use the above-mentioned energy optimisation protocols (non-IP) (e.g., *Zigbee*) and only communicate within their local network.

From these characteristics, it follows that the data needs of the Internet of Things are completely different from the current Internet. In the IoT, most communications will be brief asymmetrical machine to machine exchanges with many more data flowing in one direction (i.e., from the sensor to the server) than the other. In most cases, the loss of individual messages is tolerable. Lastly and most importantly, traditional peer-to-peer IP exchanges block much of the potential wealth of the Internet of Things. Large data streams will be flowing, many of which will be unknown and unplanned. Only a publish/subscribe architecture is capable of taking advantage of this knowledge by discovering interesting relationships and data flows. And only a publish/subscribe network can scale to the enormous size of the Internet of Things [14].

### 2.3. Management and Identification of Devices

Identifying things presents a particular challenge. Today devices can be identified through different maps (e.g., Unique Identifier, UID, or level of communication identifiers such as, Medium Access Control, MAC, and IP addresses). In order to find information and services related to things, it is necessary to have a resolution infrastructure which links identifiers of things to other devices on the network. Probably the clearest example of such infrastructure is the so called Object Name Service (ONS) in the Electronic Product Code (EPC) global network [22].

### 2.4. Sensor Modelling. The Semantic Web

Beyond identifying things, different applications must be able to access and use the information coming from things. This goal is difficult to achieve due to the heterogeneity of data representation formats. There are a number of initiatives and standardisation bodies in the field of WSN. IEEE 1451 [23] is a set of smart transducer interface standards. The Open Geospatial Consortium Sensor Web Enablement (OGC SWE) [24] has created three different sensor modelling standards and observations: Sensor Model Language (SensorML), Transducer Markup Language (TransducerML) and Observations & Measurements (O&M). Standardization of OGC SWE is a revolutionary approach to the operation of Web-enabled sensors. The aim of SWE is to create web-based sensor networks (*Web of Things,* WoT) making all sensors and sensor data repositories *discoverable, accessible* and, where applicable, *controllable* through the *World Wide Web.*

Another obstacle standing in the way to interoperability is the lack of interconnection standards between wireless networks (e.g., between *Bluetooth* and *Zigbee*).To meet that challenge, the OGC and the *World Wide Web Consortium* (W3C) [25] have been conducting research and doing standardisation work following a data-centric approach [26]. The term Semantic Web refers to W3C’s vision of the Web of linked data. Semantic Web technologies allow developers to create data stores on the Web, build vocabularies, and write rules for handling data. The Semantic Sensor Web (SSW) [27] is an approach in which semantic metadata is registered along with sensor data to provide context awareness [28]. Specifically, OGC SWE-based spatial, temporal and thematic metadata is registered.

### 2.5. Architecture

The multiplicity of solutions to address each of the challenges posed provides an indication of the current landscape of standardisation. Currently, the main IoT architectures are:

- The European Telecommunications Standards Institute M2M Technical Committee [29].
- International Telecommunication Union—Telecommunication sector view [30].
- Internet Engineering Task Force architecture fragments [31].
- Open Geospatial Consortium architecture [31].
- Internet of Things-Architecture (IoT-A) [32].

The IoT-A differs from the rest in that it creates a reference architecture consisting of a horizontal ARM (*Architectural Reference Model*) enabling the design of any IoT application. IoT-A and FIWARE are both European initiatives. Though they are in many aspects independent to each other, FIWARE usually follows the model defined by the IoT-A. Despite these efforts, there is still no architecture for IoT systems, nor is there a widely recognised universal set of standards.

### 2.6. The Need for Middleware and Open Interfaces

Middleware is the cornerstone of any IoT system as it is crucial for efficient machine to machine communication insofar as it masks the heterogeneity of devices, communications and networks. Middleware provides a set of enablers such as standardised Application Programming Interfaces (API), protocols and infrastructure services to support the swift and accurate development of services and distributed applications of Future Internet services.

In light of their importance, many research projects have focused on IoT middleware, the latter being studied, compared and classified by different authors [33,34]. In most cases, WSN middleware is implemented as middleware embedded in the node. A list of WSN middleware, software/OS and WSN programming languages is available at [35]. Each middleware solution focuses on different challenges posed by IoT such as data volume management, device management, interoperability, context awareness and many more. A middleware solution that addresses all the challenges posed by IoT has yet to be designed.

The current trend in middleware development is the use of a Service Oriented Architecture (SOA) services oriented approach and the implementation of Web standards (SOAP and RESTful) [35,36]. By extending the Web paradigm to devices, the latter can become a natural component in the construction of any IoT application and facilitate the integration of device services into any business system that is based on SOA. IoT applications can thus become independent of technology and programming language. This will help drive the IoT applications development market, Open APIs being a key component in its establishment.

WSO_2_ [37], PubNub [38] and FIWARE [39] are clear examples of this new type of platform. Specifically, FIWARE applies the concepts of the SENSEI project [40] and of IoT-A architecture [41]. Reference [1] describes the evolution of *Farm Management Systems* from monolithic systems to open systems capable to operate over the Internet. This paper shows a broad range of possible scenarios in the precision farming domain, where FIWARE technology can be applied, and it also offers a general description of FIWARE technology. However, it does not describe in detail how to use the FIWARE components to develop precision farming applications, or the performance and scalability of the resulting applications. Reference [42] complements and expands the contents of reference [1]. This paper shows a deployment of the FIWARE technology that represents a concrete example of the use of FIWARE in precision agriculture: a greenhouse. This paper presents the system components at a very high level of abstraction without entering into the details of application development. In addition, this paper is mainly focused in the cloud aspects of the farm management systems.

### 2.7. Cloud Computing and IoT

The cloud computing paradigm is one of the most positive aspects of ICT development for the IoT insofar as it allows independent and virtualized execution environments to host multiple applications in an isolated fashion on the same hardware platform, typically in large data centres. Additionally, cloud computing has the advantage of providing interconnection to different businesses if they are running on the same platform.

Cloud computing is also a key enabler when moving from a product-centred to a services-centred approach due to its elasticity that allows companies a “pay-as-you-grow” offer.

Closely related to the issue of data centres, intelligence software and data processing will play an increasingly important role in IoT solutions. A popular concept today is *Big Data* which refers to the growing number and size of data sets available to businesses and individuals for compilation and analysis. New paradigms have emerged in the field of Big Data. *MapReduce* [43] is one that has generated high expectations in scenarios where latency is not a critical requirement. Naturally, these technologies are therefore key IoT enablers allowing the aggregation and collection of massive sets of data that devices and sensors are able to produce. The IoT is unique compared to other Big Data applications such as social network analysis because, as described above, communication between machines is different from communication between humans.

## 3. FIWARE

### 3.1. Description

The FIWARE project is being developed as part of the *Future Internet Public Private Partnership* programme [44] launched by the European Commission in collaboration with the information and communication technology industry.

FIWARE is designed to be the core of the *Future Internet* covering all application domains. Therefore, FIWARE architecture is very extensive and is broken down into seven completely open technical chapters [45] based on components known as *Generic Enablers* [46] which offer reusable and configurable functions across multiple application domains.

This section reviews those aspects of FIWARE that facilitate the understanding of this work. We will begin by introducing concepts related to modelling and information management. Subsequently, an overview of FIWARE’s IoT architecture will be given.

### 3.2. FIWARE NGSI Context Management

FIWARE specification is based on the Open Mobile Alliance Next Generation Service Interfaces (OMA NGSI) Context Management [47] standard to manage and exchange information.

#### 3.2.1. Context Information Model

All information handled by applications is modelled through the definition of a Context Information Model composed of contextual elements which we will refer to as *entities*. Entities may refer to physical objects (i.e., sensors or actuators), to hardware and software (i.e., an IoT programme) and to high level abstractions that model *things* and group both physical objects and *resources*.

**Figure 1:**
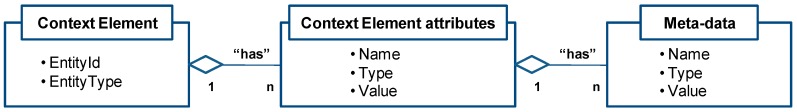
Context element conceptual diagram.

Entities are represented by the generic data structures shown in Figure 1. Entities are uniquely identified by an Entity Id/ Entity Type pair. Entities have *attributes*, characterised by a triplet (<name_attribute, type, value>). And each attribute can have optional *semantic data* (metadata) also characterised by triplets <name_metadata, type, value>. The values of the attributes become the system’s contextual information. A fundamental concept is that entities are not linked to a specific representation formalism. For example, they can be represented as an XML document (SensorML, ContextML, etc.) or as entries in a NoSQL database (MongoDB). An important advantage of this conceptual model is its compatibility with IoT formats (SensorML) while also allowing additional extensions.

#### 3.2.2. Events

In FIWARE-based IoT systems, *events* refer to something that has happened in the system. Changes in contextual information, (update of already existing entities and the creation of new ones), are considered events. FIWARE is an event-driven architecture and Generic Enablers are defined taking this fact into account [48].

#### 3.2.3. FIWARE NGSI API

The OMA NGSI-9 and NGSI-10 interfaces are HTTP RESTful APIs [45]. The FIWARE versions of these APIs allow mediation between a large number of services. The NGSI-9 interface helps makes the contextual information produced by an entity accessible to other entities. In so doing it provides two operations (among others): *registerContext* to register the entities that publish their data and *discoverContextAvailability* to discover them. The NGSI-10 API enables the contextual information exchange itself. To do that it offers, among others, *updateContext* operations to update contextual information and *subscribeContext* to create subscriptions to contextual information.

#### 3.2.4. FIWARE NGSI Associations

*Things* are a special type of *entity*. Things are abstractions of the real world and often pool *resources* according to their *location* and *attribute type*.

An *association* [49] is an ordered pair between a device/resource-level entity and a thing-level entity. Its purpose is to enable a transition from device/resource-level data to thing-level data and vice versa. Thus, associations describe relationships between these two levels of abstraction.

#### 3.2.5. Attribute Domains

An *attribute domain* is a set of closely related context attributes. Each attribute has a name and belongs to a single domain. Domains in an NGSI operation are very useful because the attributes in that domain are always requested, provided, updated or stored at the same time. This implies that the creation and updating of data within a domain are atomic and that context data associated with their attributes are always consistent.

### 3.3. IoT Architecture

**Figure 2:**
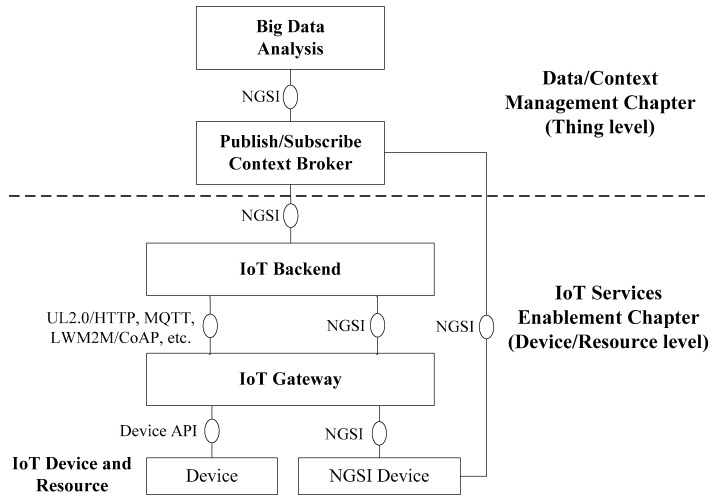
FIWARE IoT architecture.

FIWARE IoT architecture (Figure 2) is the result of the composition of the Generic Enablers of the IoT Services Enablement [50] and Data/Context Management [51] chapters. This is a flexible architecture able to address the challenges discussed in Section 2. From a physical standpoint, the IoT Services Enablement chapter defines three domains: (1) IoT Device and Resource, (2) IoT Gateway and (3) the IoT Backend, which are briefly described below.

#### 3.3.1. IoT Device and Resource

A *device* is a hardware component that measures properties of its environment or acts on that environment. Therefore, sensors and actuators are devices. On the other hand, IoT *resources* are computational elements (software) that allow devices to carry out detection or operational tasks. Generally, IoT resources are hosted on the device. There are two types of devices for FIWARE depending on whether or not they use FIWARE NGSI middleware. Devices that are not equipped with FIWARE NGSI middleware do not have the capacity to accommodate the computational resources required to provide NGSI services.

#### 3.3.2. IoT Gateway

Gateways concentrate information from end devices and channel it to consumers. This is hardware generally found near the end devices (sensors/actuators). Gateways also provide computational resources for devices within their area of influence. Just as with devices, a distinction must be drawn between two types of gateways depending on whether or not they host FIWARE middleware. The gateways that do not host FIWARE can be integrated into the system by connecting to FIWARE gateways. FIWARE gateways host two GEs: Protocol Adapter and Data Handling [46,50]. The Protocol Adapter abstracts the communication protocols used by devices (e.g., Zigbee, CoAP [2,35]) by translating them to the FIWARE NGSI protocol. Data Handling is the first stage of intelligence intended to process data in real time. To do this, it collects information from the Protocol Adapter that compile raw data from the sensors, transforming them into relevant events and then propagating them to the IoT Backend.

#### 3.3.3. IoT Backend

This is a cloud environment that hosts one or more of the following Generic Enablers: IoT Discovery, IoT Broker and Backend Device Management [46,50]. IoT Discovery allows device/resource discovery after registration/announcement of its availability (analogy: “yellow pages”). It also stores *associations* between devices/resources and the things of an IoT system. The IoT Broker interacts with the Publish/Subscribe Context Broker GE (Figure 2) and the Data Handling GE inferring the status of the *things* from *devices*/*resources* or vice versa through communication with IoT Discovery. Lastly, Backend Device Management allows for management of context entities associated with devices and provides several communication protocol translators (IoT agents) such as Ultralight 2.0 over Hypertext Transfer Protocol (HTTP), Message Queuing Telemetry Transport (MQTT) or OMA LWM2M/IETF Constrained Application Protocol (CoAP), among others. The IoT Backend communicates with end devices using standard protocols (MQTT [2,35], CoAP, etc.) or through the FIWARE NGSI protocol, connecting either through IoT gateway(s) and/or direct interfaces. From a more abstract point of view, beyond physical entities and computation resources, the Data/Context Management chapter defines the following Generic Enablers:

#### 3.3.4. Publish/Subscribe Context Broker GE

The mission of this GE is to achieve the total decoupling of producers and consumers of context information. To do this it implements the Publish/Subscribe [52] design pattern. Context producers (devices, resources and things) publish their data in this GE without needing to know who the consumers of such data are. For their part, consumers of context data do not need to know who produced it. In other words, they are simply interested in the event itself, not who generated it. As a result, the Publish/Subscribe Context Broker GE [46,51] (Context Broker hereafter) is an excellent bridge allowing external applications to manage events related to the Internet of things in a very simple way by concealing all the complexity of data compilation. To achieve this, the Context Broker uses the standard interfaces NGSI-9 and NGSI-10 which enable the recovery of said information from context producers (i.e., IoT Backend or NGSI device) to consumers of context (i.e., Big Data Analysis GE).

#### 3.3.5. Big Data Analysis GE (Cosmos)

The goal of this Generic Enabler [51] is to deploy the means with which to analyse batch data in order to reveal new information. In this type of processing, batch data is stored in advance and latency is not extremely important when they are processed. We would note that today the batch processing block has been developed to a large extent in Cosmos [46] (reference implementation of Big Data Analysis). This block integrates the Hadoop [53] ecosystem that includes the MapReduce paradigm. Also, Cygnus-NGSI [54] is part of the Big Data Analysis GE ecosystem (Cosmos). This is a connector designed to provide persistent storage of context information from the Context Broker in external repositories. In other words, the Context Broker only stores the last value referring to the attributes of each context entity. Therefore, if an IoT application requires access to historical context information, persistence in some other storage is required, value for value, using Cygnus-NGSI.

Internally, Cygnus-NGSI is based on Apache Flume [55], a technology that addresses the design and implementation of collection agents and data persistence. In fact, Cygnus-NGSI is a Flume agent essentially consisting of a source, a channel and a sink. SmartPort is a platform that covers a demanding need for big data analysis based on FIWARE [56].

## 4. Testbed

The purpose of the testbed is to measure the performance of the FIWARE platform. For practical reasons, it has been deployed in a laboratory environment. However, an IoT use case scenario has been conceived and subsequently analysed to identify the design requirements for the desired testbed.

### 4.1. IoT Use Case Scenario

**Figure 3:**
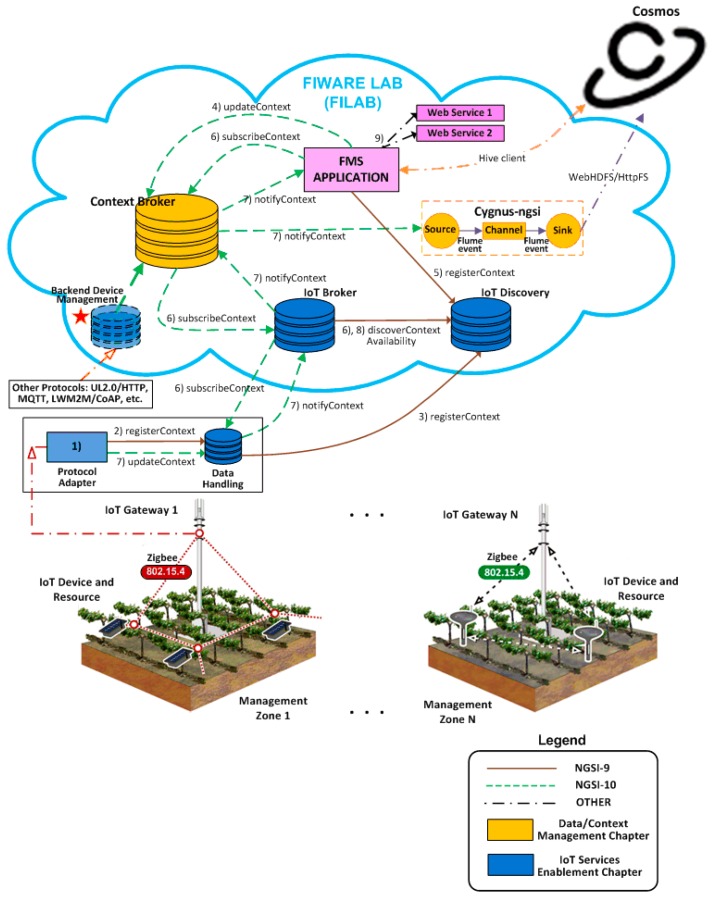
FMS envisioned based on FIWARE IoT architecture (lower part drawings are from [57]).

We are assuming a distributed scenario in the field of precision agriculture/viticulture (i.e., crops in several different regions or countries). Hypothetically, this is a complex IoT scenario as it involves managing a large number of *resources* associated with sensors and actuators. Specifically, the sensors detect data relating to the environment (e.g., air temperature, relative humidity, solar radiation and precipitation) and the state of the vineyards (e.g., water and nutrients). The actuators are installed in crop irrigation systems. The system has to be scalable: if the number of sensors and actuators is increased, only computational resources would need to be increased without implying greater complexity or system redesign. To address this challenge of scalability, the implementation of the *Farm Management System* (FMS) illustrated in Figure 3 is planned.

The FMS that has been designed takes advantage of the new features offered by the *Future Internet* by relying on the IoT FIWARE architecture (see Section 3.3). Vineyards are divided into management zones, each with a Zigbee wireless sensor network (WSN). The IoT Gateways of these networks host characteristics of Protocol Adapter and Data Handling GEs. These gateways are located in the vicinity of the devices. The IoT Gateways connect to a specific FILAB account [58] (FIWARE testbed), which hosts instances of IoT Broker, IoT Discovery and Context Broker GEs. Our application, FMS, is also hosted in this instance. The functions performed by these components are explained below.

**Registration of IoT Devices and Resources**
  1. When starting the system, the Protocol Adapter automatically detects the IoT *resources* associated with the devices, sensors or actuators in its management zone.
  2. For each *resource* detected, the Protocol Adapter sends a *registerContext* request to Data Handling.
  3. When the Data Handling register receives the *registerContext* request, it creates an *entity* in its local database *modelling the resource*, and sends a *registerContext* request to IoT Discovery. IoT Discovery bundles information on *resources* from different management zones.
**High-Level Modelling of Things**
  4. We now turn our attention to the opposite end of Figure 3, the application of FMS and the Context Broker component. The FMS application implements a RESTful HTTP client that can invoke Context Broker services. The *updateContext* service of the Context Broker is invoked both to create new *entities* that model *things* and to update the status of existing entities. Two types of entities are thus created: the resources that are detected automatically (steps 1 and 2) and the things which are defined explicitly from the application. In FIWARE, resources and things represent different levels of abstraction, as also shown in Figure 2. Things tend to pool resources according to their location and type of attributes. The Context Broker can access information from the resources through a component not yet presented: the IoT Broker.
  5. Mapping between resources and things is achieved in IoT Discovery which records *associations* between entities that model things and those that model system resources.
**Subscription to the Information Generated by the Entities**
  6. Once entities have been defined, whether these be resources or things, any application can subscribe to the information that these entities generate by invoking the *subscribeContext* operation from the Context Broker. In our case the FMS application creates entities and subscribes to them, and Cosmos, which is a storage system for FIWARE Big Data, have also been subscribed to them. The Cosmos connection is not direct but rather through another FIWARE component, the injector Cygnus-NGSI. Note that there is a chain of subscriptions and brokers (Data Handling, IoT Broker and Context Broker). The most important is the Context Broker which makes things and resources accessible to applications and is the reference implementation for the system’s other brokers. The Context Broker subscribes to the context information of the IoT Broker which mediates between the Context Broker and different Data Handling associated with different areas or management zones of the crop field. The IoT Broker uses the *discoverContextAvailability* service of IoT Discovery in order to discover the resources associated with the things with which the Context Broker works. For our case study, all subscriptions are *onChange*: only events are generated and therefore notifications are sent to subscribers when changes occur in entity attributes.
**Information Notification**
  7. Crop data is acquired by IoT devices and resources that transmit this information to the IoT Gateway where they are received by the Protocol Adapter. In Figure 3 devices use the IEEE 802.15.4/ZigBee protocol but could use others (CoAP or RFID [35]). In fact, Protocol Adapters hide communication protocols with physical devices from the rest of the components. The Protocol Adapter transmits the information received to Data Handling through an *updateContext*. Data Handling then notifies (*notifyContext*) the IoT Broker which, in turn, notifies the Context Broker which, in turn, passes it on to its subscribers, in our case the FMS and Cygnus-NGSI application.
  8. The case of the IoT Broker is special because it must interact with the IoT Discovery component to translate notifications at resource level to the corresponding notifications at thing level sent to the Context Broker. The Cygnus-NGSI injector is another special case which is explained below.
  9. Finally, the FMS application implements two Web services from the information it is supplied by the Context Broker and Cosmos. In the first service, information is published in real time as are specialised reports on the environment and status of vineyards (e.g., pest prediction) thus facilitating the taking of decision concerning irrigation, fertilisation and pest control. The second Web service is a human machine interface (HMI) enabling the remote activation of the control elements installed in crop irrigation systems. In this case, information travels in the opposite direction, the first step being the sending of control commands to the Context Broker.

In short, the FMS is context aware: the information gathered from the sensors is acquired, modelled and compiled for processing following analysis and *knowledge* extraction. This *knowledge* facilitates decision-making and the consequent sending of activation commands to the actuators. This is possible thanks to the orchestration of IoT services provided by FIWARE allowing *resources* associated with *devices* to become *searchable, accessible* and *usable* thus maximising their interaction with the application of FMS. Thus, in the architecture (Figure 3) the Context Broker interoperates with the IoT broker and the IoT Data Handling that implement Publish/Subscribe components with characteristics similar to the former. Thus, a network of *brokers* is created that allows uncoupled interaction between services that produce and consume contextual information. This is an example of the federation concept adopted by FIWARE from the IoT-A architecture (see Section 2.5).

This scenario could be more complex if the physical devices used communication protocols not supported by the Protocol Adapter (e.g., MQTT). In this case the deployment of a Backend Device Management instance (GE labelled with a star in Figure 3) would be needed to address this interoperability. It can therefore be concluded that the Context Broker handles all the system’s contextual information, mediating between the elements that produce that information (IoT Broker, Backend Device Management and implementation of the FMS) and the elements that consume it (IoT Broker, Backend Device Management, implementation of the FMS and Cosmos through Cygnus-NGSI). Hence, the Context Broker is the backbone of the system’s data flow. The Context Broker plays a decisive role in any context aware system based on FIWARE technologies. That is why a testbed has been designed to assess its performance.

### 4.2. Design

The following requirements had to be met in the design phase of the testbed:

- Initially oriented towards FIWARE but adaptable for use with other platforms thus enabling comparative studies.
- Oriented towards measurement of performance and data storage capacity but extendible to include other properties.
- Establishment of data generation parameters: number of IoT nodes, data frequency generation and payload.
- Establishment of test parameters in FILAB: number and characteristics of virtual machines (VMs) deployed, number and characteristics of Generic Enablers instantiated and parameters of the Cygnus-NGSI injector.
- Test settings and autostart against FIWARE instances.

**Figure 4:**
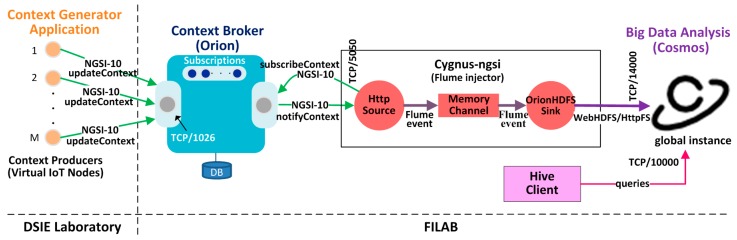
A testbed to evaluate the performance of the FIWARE platform.

The testbed designed (Figure 4) takes the most important elements of the case study shown in Figure 3 into account, including (1) a context generating application, (2) instances of reference implementations of the Context Broker (Orion) and Big Data Analysis (Cosmos), (3) a client application (Hive) that acts as the FMS application and (4) the Cygnus-NGSI injector.

The context generator application follows the client/server architecture and runs on 13 machines located in one of the laboratories of the research group (Electronics Engineering and Systems Division, DSIE) of the Universidad Politécnica de Cartagena. This application simulates the generation of context information from an IoT node network by implementing RESTful (NGSI) clients. One design criterion is that each IoT node simulates a producer of context that can refer to an intermediate node (e.g., IoT *Broker*) or a final NGSI node (see Figure 2) that can integrate multiple sensors. In these cases, requests to the *Context Broker* are transmitted directly to update the context corresponding to entities that can be modelled on future IoT systems, posing the challenge of scalability discussed in the previous section.

The Http source of the Cygnus-NSGI injector is responsible for receiving events from the *Context Broker,* transforming them into Flume events [55] and injecting them into the channel. The Cygnus sink *(Orion HDFS Sink)* extracts the Flume events from the channel and uses the API WebHDFS/HttpFS [53] to achieve persistence of the context information contained in the body of the Flume events in the *Hadoop Distributed File System* [53] set up in Cosmos, creating a historical view of such data. Also, the application of FMS implements a Hive client [53] for the execution of *MapReduce* tasks associated with new generation mining and analysis techniques (reasoning models) that process historical context information stored in Cosmos. The resulting perceptions are extracted through *HiveQL* language.

### 4.3. Experimentation Environment

There is a community account in FILAB’s Spain2 cloud infrastructure enabling the deployment of a virtual machine (VM) hosting an instance of *Orion Context Broker* (version 1.2.1), version 3.0.12 of a MongoDB database and version 0.13.0 of the Cygnus-NGSI injector. The VM has the following characteristics: 4 VCPU, 8 GB of RAM and 80 GB disk. This more than meets the hardware requirements of Orion and Cygnus-NGSI. An account was created in the global instance of Cosmos whose initial quota of 5 GB of storage is expandable. DSIE laboratory machines have the same characteristics:

- Hardware: HP Compaq Business Desktop dc7700p (Intel Core 2 Duo E6600/2.4 GHz Dual-Core processor, 4 MB cache per core L2 cache 4 MB and 2 GB RAM).
- Software: Windows 7 Home Premium operating system, Luna Eclipse development environment SR2 (4.4.2) and context generator application.

### 4.4. Configuration

From a performance point of view, FIWARE recommendations were taken into account to refine the following testbed com*p*onents: (1) MongoDB; (2) Orion and (3) Cygnus-NGSI. The most important aspects of configuration are as follows:

First, we used version 3.0.12 of MongoDB, recommended for intensive update scenarios. In addition, the adjustments limiting use to deployed VM resources were checked and *Transparent Huge Pages* were disabled (THP) [59].

Second, Orion parameters were adjusted for scenarios with high update/notification rates (-*reqPoolSize,* -*dbPoolSize* and -*notificationModethreadPool:q:n)* [59]. These parameters were adjusted for each test.

Third, Cygnus-NGSI components were adjusted to increase performance and avoid potential bottlenecks in order to properly assess Orion. To achieve this we opted for an HTTP source where the *OrionRestHandler* was configured to convert NGSI events from Orion into Flume events. Concerning channel design, we chose *Memory Channel* because of its performance when implemented directly in memory. This type of channel is ideal for flows that require increased performance and are willing to lose data in the case of Flume agent errors. Finally, we chose an *Orion HDFS Sink* whose performance could be enhanced by using the *batching* mechanism to reduce the number of writes in the HDFS storage configured in Cosmos. This mechanism is configured in each simulation through batch size and batch_timeout parameters [60].

### 4.5. Context Generation Methods

In each test, the application developed offers users a choice between two ways of generating NGSI traffic. The first method (blocking method) simulates intermediate IoT node NGSI traffic generation. Its implementation is supported by the *java.net* API and its functionality is based on the *HttpURLConnection* class. This class only allows blocking connections. Thus, in each simulation the same number of persistent HTTP connections as simulated IoT nodes are opened.

The second method (non-blocking method) simulates final IoT node NGSI traffic generation. Its implementation is focused on mechanisms that allow concurrent traffic generation [61]. In this case, the *SocketChannel* class of the *java.nio* API was used to create non-blocking connections. Therefore, in each simulation the same number of connections as NGSI requests generated are opened.

Another implementation detail to keep in mind is that each NGSI client (blocking or not) simulates the behaviour of a virtual IoT node in a separate thread.

### 4.6. Startup and Operation

Initially, Orion is started as a system service. Then, the context generating application is started executing 1 master process and up to 12 slave processes. The master process launches a graphic user interface (GUI) that automates the configuration and startup of the test. Test parameters are introduced in the GUI, i.e., number of IoT nodes, frequency of NGSI traffic generation and *payload* that determines the attribute domain of the virtual IoT system entities. The master process also sends *updateContext* requests to Orion to create entities. After starting Cygnus-NGSI, also implement *onchange* type *subscribeContext* request sender and the generation and broadcast transmission of a stream of test parameters through the network.

Each slave process decodes the steam, synchronizes with the master process and executes one of the context generation methods in a certain number of NGSI clients. As a result, M virtual IoT nodes send context information to Orion through the TCP/1026 port. When an attribute of an Orion database *entity* is updated, an *event* is generated and a notification is sent to Cygnus-NGSI through the TCP/5050 port.

Finally, the Cygnus-NGSI injector handles information persistence in Cosmos through the TCP/14000 port. At the conclusion of the simulation, the master process compiles all the measurements from the slave processes and performs additional operations to obtain performance statistics for the experiment. These statistics are presented to the user through the GUI and stored in a text file formatted for uploading to the *RStudio* development environment. The Hive client allows queries of historical information stored in Cosmos through the TCP/10000 port.

### 4.7. Test Plan

Considering the *payload* of the *updateContext* requests sent to Orion, four types of tests were run (1 kB, 10 kB, 100 kB and 1 MB). Each of these tests is divided into two categories depending on the context generation method used (blocking or non-blocking). The first category includes performance tests employing the blocking method to measure throughput parameters (expressed in requests per second or kilobytes per second) and round-trip time (in milliseconds). The second category includes performance tests employing the non-blocking method to measure the throughput parameter (expressed in requests per second or kilobytes per second). 20 simulations were launched per category on different days and in different time slots. The test plan, therefore, involves more than 400 simulations. The following section shows the representative results of each type of test.

The following procedure was followed in the tests. The IoT nodes are modelled by threads associated with processes. In the initial simulations, we started with 12 single-threaded processes, each executed on a different machine. When the number of IoT nodes exceeds 12, new threads are started in the processes. To simplify testing, multiples of the number of processes started for the tests are simulated, generally 12, in order to equitably distribute the simulation load among the processes. In certain cases, to refine the results, we worked with fewer machines and therefore fewer processes (always one process per machine). That is why the graphs show measurements with a number of nodes that is not a multiple of 12. A simulation concludes when each virtual IoT node has tried to send 200 requests to the Orion Context Broker. The throughput and round-trip time values of the simulation are calculated from the average of the 200 measurements obtained.

## 5. Results

In order to evaluate the performance of the Orion Context Broker, throughput and latency were plotted graphically depending on the degree of concurrency simulated in each type of test. To study the behaviour of the *Orion* RESTful server numerous simulations were made. The realization of these simulations was a very time-demanding task due to network latency. The results obtained are not exact. They do, however, offer a realistic view of the performance of this *Generic Enabler* under different deployments and load conditions.

**Figure 5:**
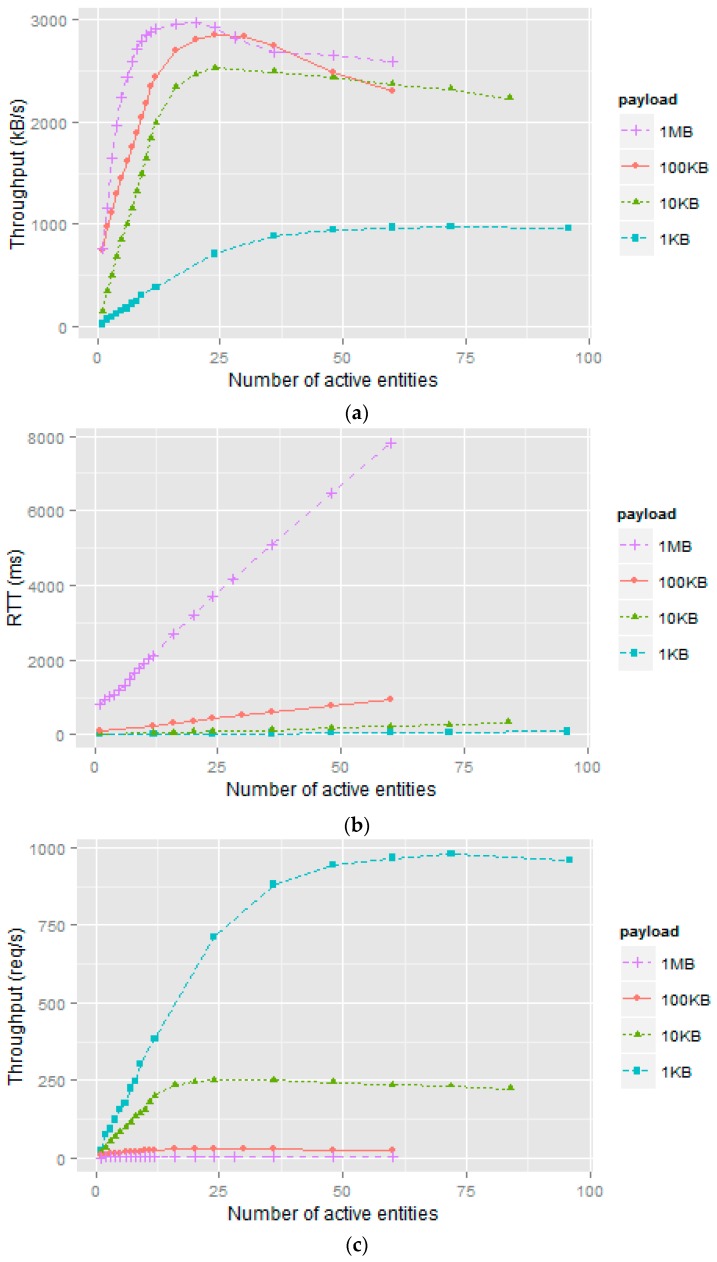
Results obtained using the blocking method: (**a**) Higher data transfer rate at high loads by few nodes; (**b**) The system exhibits increased latency as load and concurrency (number of active entities) increase; (**c**) Better performance with respect to the number of requests served and worse with respect to payload.

Figure 5 illustrates the results obtained when using the blocking method to generate the context requests for each *payload* established in the test plan (1 kB, 10 kB, 100 kB and 1 MB). A new message is sent as soon as the previous one has been completely delivered. Figure 5a–c illustrates throughput (Th) measured in terms of kilobytes per second (kB/s) or requests served per second (req/s) and latency expressed as Round-Trip Time (RTT) measured in milliseconds (ms) versus number of NGSI clients, which can be sensors (edge devices in general) or intermediate nodes. Simulations results will be interpreted differently depending on whether the active entities are sensors or intermediate nodes. For now, results are presented and later they will be discussed. Three ranges of interest can be distinguished in these graphs as shown in Table 1, Table 2 and Table 3. Tables are included to show clearly the points of interest without overloading the figures. These ranges provide information on the behaviour of the Orion Context Broker. The graphs show that Orion has an initial range (close to linear) where throughput increases at a more or less linear rate and RTT remains constant or linear. The second range of interest (increase slow/fast) corresponds to a gradual increase in throughput up to its maximum value while RTT rises at a swift pace. From the point of maximum throughput, system performance declines as payload and concurrency parameters increase, resulting in the third and final range of interest (degrading performance). In this range, the above parameters tend to increase RTT (waiting time) and decrease throughput (requests processed). This was the expected result but it is interesting to see where these changes occur, the most useful reference being the point of maximum throughput.

To analyse the performance of Orion in greater detail, we studied the maximum throughput values obtained in each test type. This parameter can be evaluated from two perspectives: Data transferred and requests processed. From the first perspective (data transferred, see Figure 5a and Table 2):

- The highest rate of data transfer was 2963 kB/s obtained when simulating a payload of 1 MB and 20 NGSI clients (see Table 1). RTT in this simulation is 3200 ms (see Figure 5b).
- The second best result was 2846 kB/s obtained when simulating a payload of 100 kB and 24 NGSI clients. However, RTT was 430 ms, clearly much lower than the preceding case.
- The next throughput measurement in descending order was 2520 kB/s corresponding to the simulated payload of 10 kB and 24 NGSI clients. Although the same number of active entities as in the previous case was used, RTT was lower (83 ms).
- Finally, the lowest data transfer rate (978 kB/s) was obtained with the simulation of a payload of 1 kB and 72 NGSI clients. We should note that a much higher number of active entities was needed in comparison with the other simulations. Also, RTT (72 ms) is the lowest value of the cases included in this analysis.

In other words, the system performs better when faced with high loads generated by few nodes. The increase in the number of nodes causes a very sharp decrease in the volume of data that can be transferred. From the second perspective (requests served per second, see Figure 5c and Table 3):

- The lowest rate of requests processed was 2.89 req/s with a payload of 1 MB.
- The second worst result was 28.46 req/s at a payload of 100 kB.
- The next result (252 req/s) at a payload of 10 kB is substantially better.
- Finally, the measure of throughput obtained (978 req/s) in the simulation of a 1 kB payload is far superior to the other cases in the study.

We would note that here the behaviour is exactly the opposite of the preceding case. The system performs better in terms of the number of requests and worse with regard to payload.

To check whether a greater throughput number in terms of requests per second is possible, we ran a test with a minimum payload of 300 bytes. The results are similar to those in the case of a 1kB payload. For that reason they are not shown here. No significant improvements were found below a payload of 1 kB.

Scenarios that were densely populated with sensors such as the one discussed in Section 4.1 imply a high update rate. For this reason, a RESTful server that supports a high degree of concurrency and which can also process a large number of applications nearly in real time is required. The results described show that the best choice to get the most out of Orion in this type of scenario is a payload of between 1 kB and 10 kB. The final payload depends on the needs of each IoT application (estimated degree of concurrency or number of active entities, attribute domain, etc). An interesting scenario is to study the behaviour of FIWARE faced to blocking and non-blocking requests.

Blocking requests are typical of intermediate nodes, especially the connection between the Generic Enablers IoT Broker and Context Broker (see Figure 3). Non-blocking requests are typical of sensors, or IoT final nodes, which for energy efficiency reasons can not be blocked waiting for a response from the server. Normally, a sensor sends an asynchronous request and closes the connection to save energy. This simulates the behaviour of a final IoT node which does not maintain the connection, when unnecessary in order to conserve energy.

To simulate NGSI traffic generation from final IoT nodes, the non-blocking context generation method must be used so as to permit the generation of NGSI traffic in an asynchronous manner, with load peaks (bursts) that exceed the capacity of the Orion server. The purpose of this test is to measure maximum throughput in terms of requests served per second or kilobytes per second for both type of requests, blocking and non-blocking. The tests were performed with a payload of 1 kB, i.e., that which allowed simulation of the highest degree of concurrency with the blocking method. Thus, blocking and non-blocking simulations were conducted with the optimum load parameter for the blocking case. This facilitates comparisons.

**Figure 6:**
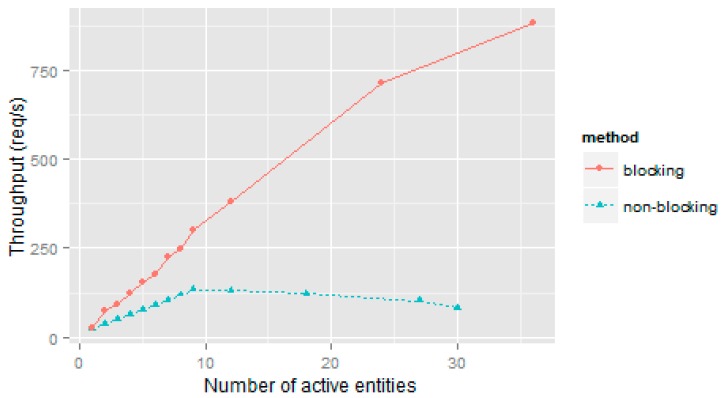
Throughput obtained using the blocking and non-blocking methods.

Figure 6 illustrates the throughput in blocking and non-blocking conditions. In the case of blocking requests, every blocking request is issued in an independent thread. In this way, many simultaneous blocking requests can be simulated. In the case of non-blocking requests, each non-blocking socket opens a connection to Orion every 50 ms. As the graph indicates, Orion has an initial range in which throughput increases almost linearly up to a maximum of 133.2721 requests or kilobytes per second at a concurrency of 9. As from this value, Orion enters overload reducing its throughput as concurrency increases. The simulation of 30 non-blocking sockets is a clear indication of how this state affects throughput (83.4611 req/s).

If we compare the maximum throughput measured when traffic is generated by means of the two testbed methods, we see that the value obtained with the non-blocking method is significantly lower than that obtained with the blocking method. We would note that in the blocking context generation method, each NGSI client generates an *updateContext* request once the previous one is completed. Clients remain in sync with the server and the rate of requests generated never exceeds the capacity of the Orion server.

## 6. Conclusions and Future Work

This paper has shown a use case of FIWARE with enough detail level to provide an accurate view of how a FIWARE application is developed. It is a complex technology as it is designed to adapt to the variability of a wide range of applications, which demand different interaction schemes. At the same time, FIWARE assumes applications driven by events, which may show an asynchronous behaviour. All these features inevitably imply the use of many components with interdependencies. The FIWARE architecture, which defines these dependencies, is based on common design patterns in the communications software, mainly the use of a broker. The use of common design patterns makes both the understanding of FIWARE technology and its use easier for application developers. However, in our opinion, the documentation is not well organized. Furthermore, it does not always offer enough detail level to give a specific answer to an implementation problem. The deficiencies in the documentation involve investing a lot of time and effort into solving relatively simple problems.

In this regard, we believe that, this work, without going into coding details, provides a useful guide to identify the components needed to develop a FIWARE application as well as the interfaces used to connect them.

Regarding the performance of the resulting applications and based on the results obtained in the testbed, it is safe to say that the choice of payload is critical in the performance of the Orion Context Broker. As described in Section 5, an optimal payload maximises Orion’s performance in terms of throughput and latency. Furthermore, that payload allows Orion to support a greater degree of concurrency.

Precision agriculture applications, like many other IoT applications, are characterised by two types of situations: (1) very frequent exchanges of small amounts of data from low capacity sensors; (2) mass transmission of off-line data, i.e., data obtained from a drone or a camera mounted on an agricultural vehicle. FIWARE performs well in both cases.

For all of the above, we conclude that the FIWARE platform meets the requirements of today’s IoT applications in the sphere of precision agriculture.

In other domains there are applications that require the transmission of massive volumes of data in real time (streaming in real time) accompanied by the processing of such data online as they are generated. Apart from the Orion Context Broker, FIWARE also supports other Generic Enablers [46] as Kurento, Complex Event Processing (CEP) and Cosmos (Stream Processing) to implement solutions in such scenarios. It would therefore be interesting to check the performance of FIWARE in this type of scenario (case 2).

In the case of future IoT scenarios, Section 4.1 provided a description of a Farm Management System (FMS) composed of the FIWARE elements discussed in Section 3.3. The FMS addresses the challenges of scalability and interoperability that were discussed in Section 2. On the one hand, the Generic Enablers Protocol Adapter and Backend Device Management are responsible for concealing communication protocols from the rest of the components with physical devices (Zigbee, MQTT, CoAP, etc). On the other hand, the Orion Context Broker is the most important component because it handles all the system’s contextual information, mediating between the elements that produce such information and those that consume it. Regarding scalability, two dimensions can be considered in the Orion Context Broker:

- Scalability in the number of *entities*. In this case, the critical (scarce) resource is the database (DB), pointing to the need to scale the MongoDB layer. The usual procedure to scale MongoDB is through the use of shards described in the official documentation of MongoDB.
- Scalability in operation requests to manage entities. In this case, additional Orion Context Broker instances can be used (each running on a different VM), plus an additional VM ahead of them running the software load balancer [46] to distribute the load between Orion nodes.

As for data persistence in Cosmos through Cygnus-NGSI, in the same way that an Orion processes farm can be created, additional Cygnus-NGSI processes responsible for receiving Orion farm notifications can be added. To do that, it is advisable to define a mapping strategy for system entities. Thus, subscriptions are established corresponding to a set A of entities that will notify the Cygnus-NGSI A process, another set of entities B which will notify the Cygnus B process, etc.

Another important feature of Generic Enabler Data Handling is the optimisation of traffic sent to the IoT Backend. This functionality helps achieve greater efficiency and reliability by using CEP techniques. In short, it is possible to increase system efficiency by injecting preprocessed context information (more relevant for the IoT application) into the backend. Thus the CEP, also discussed in the Chapter on *Data/Context Management*, would alleviate traffic sent to Cygnus-NGSI.

Summarising, it can be concluded that the combination of: (1) optimal performance tuning of FIWARE components, (2) the mechanisms provided by each component, (3) the horizontal scalability of each instance in the cloud and (4) the vertical scalability of the platform, makes the development of agricultural applications in futuristic scenarios as described in Section 4.1 possible.

Although the testbed is oriented towards measurement of performance and data storage capacity, it is extendible to include other properties. For this reason, it is possible to further the study of the FIWARE platform (analysis of case 2 above, streaming with FIWARE) or conduct a wide variety of studies focusing on current IoT middleware. In addition, all the software infrastructure that simulates the sensors and the different ways in which they generate the data (payload, data frequency generation, number of processes and threads, etc.) can be easily used to test other IoT middleware. In doing so, we will be able to compare the results obtained with FIWARE with other middleware platforms, such as WSO2, PubNub… opening up a future avenue of research through its use with other platforms enabling comparative studies.

## Figures and Tables

**Table 1:** Number of active entities simulated in each type of test.

Region	1 kB	10 kB	100 kB	1 MB
Close to linear	[1 .. 24]	[1 .. 12]	[1 .. 12]	[1 .. 8]
Increase slow/fast	[24 .. 72]	[12 .. 24]	[12 .. 24]	[8 .. 20]
Degrading performance	[72 ..	[24 ..	[24 ..	[20 ..

**Table 2:** Throughput obtained in kilobytes per second.

Region	1 kB	10 kB	100 kB	1 MB
Close to linear	[25 .. 712]	[157 .. 1996]	[749 .. 2437]	[760 .. 2640]
Increase slow/fast	[712 .. 978]	[1996 .. 2520]	[2437 .. 2846]	[2640 .. 2963]
Degrading performance	[978 ..	[2520 ..	[2846 ..	[2963 ..

**Table 3:** Throughput obtained in requests served per second.

Region	1 kB	10 kB	100 kB	1 MB
Close to linear	[25 .. 712]	[15.7 .. 199.6]	[7.49 .. 24.37]	[0.76 .. 2.64]
Increase slow/fast	[712 .. 978]	[199.6 .. 252]	[24.37 .. 28.46]	[2.64 .. 2.89]
Degrading performance	[978 ..	[252 ..	[28.46 ..	[2.89 ..
